# Parity-Related Differences in Neonatal Outcomes Among Adolescent Mothers: A Retrospective Cohort Study

**DOI:** 10.3390/children13080990

**Published:** 2026-07-25

**Authors:** Florin Mihai Sandor, Roxana Furau, Florina Buleu, Diana Camelia Bonte, Daian-Ionel Popa, Tiberiu Buleu, Izabella Petre, Oana Suciu, Cristian George Furau, Cris Virgiliu Precup, Ion Petre

**Affiliations:** 1 Multidisciplinary Doctoral School, “Vasile Goldis” Western University of Arad, 310414 Arad, Romania; sandor.florin@uvvg.ro (F.M.S.); furau.cristian@uvvg.ro (C.G.F.); 2 Department of Life Sciences, “Vasile Goldis” Western University of Arad, 310414 Arad, Romania; precup.cris@uvvg.ro; 3 Department of General Medicine, Faculty of Medicine, “Vasile Goldis” Western University of Arad, 310414 Arad, Romania; furau.roxana@uvvg.ro; 4 Department of Cardiology, “Victor Babes” University of Medicine and Pharmacy, Eftimie Murgu Sq. No. 2, 300041 Timisoara, Romania; 5 Department IV, Discipline of Biochemistry, “Victor Babes” University of Medicine and Pharmacy, Eftimie Murgu Sq. No. 2, 300041 Timisoara, Romania; 6 Research Center for Medical Communication, “Victor Babes” University of Medicine and Pharmacy, Eftimie Murgu Sq. No. 2, 300041 Timisoara, Romania; daian-ionel.popa@umft.ro; 7 Faculty of General Medicine, “Victor Babes” University of Medicine and Pharmacy, Eftimie Murgu Sq. No. 2, 300041 Timisoara, Romania; tiberiu.buleu@umft.ro; 8 Department of Obstetrics and Gynecology, “Victor Babes” University of Medicine and Pharmacy, Eftimie Murgu Sq. No. 2, 300041 Timisoara, Romania; petre.izabella@umft.ro; 9 Department of Microbiology, “Victor Babes” University of Medicine and Pharmacy, Eftimie Murgu Sq. No. 2, 300041 Timisoara, Romania; oana.suciu@umft.ro; 10 Department of Functional Sciences, “Victor Babes” University of Medicine and Pharmacy, Eftimie Murgu Sq. No. 2, 300041 Timisoara, Romania; petre.ion@umft.ro

**Keywords:** adolescent pregnancy, parity, neonatal outcomes, preterm birth, low birth weight, Apgar score, maternal age, retrospective cohort

## Abstract

**Highlights:**

**What are the main findings?**

- Parity was not significantly associated with preterm birth, Apgar score < 7, or the composite adverse neonatal outcome.
- Multiparity was associated with lower odds of low birth weight after adjustment for gestational age.

**What are the implications of the main findings?**

- Parity alone has limited value for neonatal risk stratification in adolescent pregnancies.
- Assessment should consider gestational maturity, fetal growth, and very young maternal age rather than parity alone.

**Abstract:**

**Background/Objectives**: Adolescent pregnancy is associated with an increased risk of adverse neonatal outcomes, including low birth weight, preterm birth, and impaired neonatal adaptation. While maternal age is a well-established determinant, the independent role of parity in this population remains uncertain. This study aimed to assess the association between parity and neonatal outcomes in adolescent mothers. **Methods:** We conducted a retrospective cohort study including 751 singleton live-birth deliveries to adolescent mothers aged <18 years at a Romanian secondary-care hospital. The unit of analysis was the delivery episode recorded in the institutional birth registry. Mothers were classified according to parity at the time of the index delivery as primiparous or multiparous. Neonatal outcomes included low birth weight (<2500 g), preterm birth (<37 weeks), Apgar score < 7 at 5 min, and a composite adverse neonatal outcome. Multivariable logistic regression models were used to evaluate the association between parity and neonatal outcomes, adjusting for maternal age (<15 vs. ≥15 years), gestational age, and mode of delivery, as appropriate. **Results:** A total of 751 singleton live-birth delivery episodes were included; 609 (81.1%) occurred in primiparous adolescents and 142 (18.9%) in multiparous adolescents. Parity was not significantly associated with preterm birth (aOR 0.86, 95% CI 0.48–1.56, *p* = 0.622), Apgar score < 7 at 5 min, or the composite adverse neonatal outcome. Maternal age < 15 years and gestational age were independently associated with Apgar score < 7 at 5 min. Multiparity was associated with lower odds of low birth weight (aOR 0.40, 95% CI 0.17–0.92, *p* = 0.031). **Conclusions:** Parity was not independently associated with most neonatal outcomes in adolescent pregnancies. Multiparity was associated with lower odds of low birth weight, but this finding should be interpreted cautiously because residual confounding cannot be excluded.

## 1. Introduction

Adolescent pregnancy continues to represent a complex public health issue with persistent clinical and social implications across diverse healthcare settings [1]. Although global incidence has declined in recent decades, millions of pregnancies still occur annually among girls younger than 19 years [1,2]. Adolescent pregnancy has been consistently associated with an increased risk of adverse neonatal outcomes, particularly low birth weight and preterm birth [1,2,3]. Large multicountry and population-based studies have further demonstrated increased risks of neonatal morbidity and mortality among infants born to adolescent mothers [4,5]. In addition, impaired early neonatal adaptation, including lower Apgar scores, has been reported more frequently in this population [6]. These associations reflect the combined effects of biological immaturity, including incomplete uteroplacental vascular adaptation, and social vulnerability [7,8,9]. Limited access to prenatal care, low socioeconomic status, and suboptimal health behaviors have been identified as major contributing factors [6,7]. Importantly, the magnitude of these risks varies across populations and healthcare systems, suggesting that adolescent pregnancy represents a heterogeneous clinical entity rather than a uniform risk category [9,10].

Maternal age within the adolescent spectrum is an important determinant of outcome variability. Younger adolescents, particularly those under 15 years of age, have a higher risk of adverse neonatal outcomes, including prematurity and low birth weight [5]. This gradient has been attributed to ongoing maternal growth, which may compete with fetal development for nutritional resources [11], as well as incomplete physiological and reproductive maturation [12]. However, biological mechanisms alone do not fully explain these disparities. Social determinants, including access to antenatal care and broader socioeconomic conditions, play a central role and may mediate the relationship between maternal age and neonatal risk [9].

Parity represents an additional obstetric characteristic that may influence neonatal outcomes, although its role in adolescent populations remains insufficiently defined. In adult populations, multiparity has been associated with differences in pregnancy outcomes, often reflecting physiological adaptation to prior pregnancies. However, evidence specifically addressing parity in adolescent mothers remains limited and inconsistent. Some studies suggest that nulliparous adolescents may be at higher risk of adverse outcomes [13], whereas others report no independent association after adjustment for confounding factors such as socioeconomic status and access to care [10]. These inconsistencies may reflect differences in study design, population characteristics, healthcare settings, and the extent to which important confounders, including maternal age, prenatal care utilization, and socioeconomic conditions, were accounted for in the analyses. Moreover, repeat adolescent pregnancies frequently occur in settings characterized by social disadvantage and limited access to reproductive health services, which may confound observed associations [14,15]. Consequently, whether parity represents an independent determinant of neonatal outcomes in adolescent pregnancies or a proxy for underlying contextual factors remains incompletely understood. This uncertainty highlights an important knowledge gap, particularly in settings where adolescent pregnancy remains common and is closely linked to social vulnerability.

This question is particularly relevant in Eastern European contexts. Romania continues to report among the highest adolescent fertility rates in the European Union, with marked regional and socioeconomic disparities [15]. Adolescent pregnancies in this setting are often associated with socioeconomic vulnerability and disparities in access to healthcare [13,15,16]. Despite improvements in maternal care, adverse neonatal outcomes remain prevalent among adolescent mothers. At the same time, evidence specifically examining the role of parity in adolescent pregnancies remains scarce in Romania and in Eastern Europe more broadly. Most available studies originate from different healthcare systems and sociodemographic contexts, which may limit the generalizability of their findings to populations characterized by high rates of adolescent pregnancy and unequal access to prenatal care. Consequently, evaluating parity within the Romanian setting may provide clinically relevant information and contribute to a more context-specific understanding of neonatal risk among adolescent mothers.

Therefore, the present study aimed to evaluate the independent association between parity and neonatal outcomes among adolescent mothers after adjustment for maternal age and relevant obstetric factors. Specifically, we examined whether parity was associated with low birth weight, preterm birth, Apgar score < 7 at 5 min, and a composite adverse neonatal outcome in adjusted analyses. By focusing on parity within a large cohort of adolescent pregnancies from a Romanian secondary-care hospital, this study seeks to address an important gap in the existing literature and to provide evidence from an Eastern European healthcare setting that remains underrepresented in current research.

## 2. Materials and Methods

### 2.1. Study Design and Population

We conducted a retrospective cohort study including adolescent mothers aged <18 years who delivered at the Obstetrics and Gynecology Clinic of the Arad County Emergency Clinical Hospital, Romania, between 1 January 2020 and 31 December 2024. The study was based on delivery records extracted from the institutional birth registry, and the unit of analysis was the pregnancy/delivery episode.

A total of 763 adolescent deliveries were screened for eligibility. Exclusion criteria included missing data on parity, stillbirth, and multiple pregnancy; these criteria were not mutually exclusive. Multiple pregnancies were excluded due to their distinct obstetric risk profile. Stillbirths were excluded because the neonatal outcomes evaluated in this study (birth weight, preterm birth, and Apgar score at 5 min) were defined for live-born infants.

**Figure 1:**
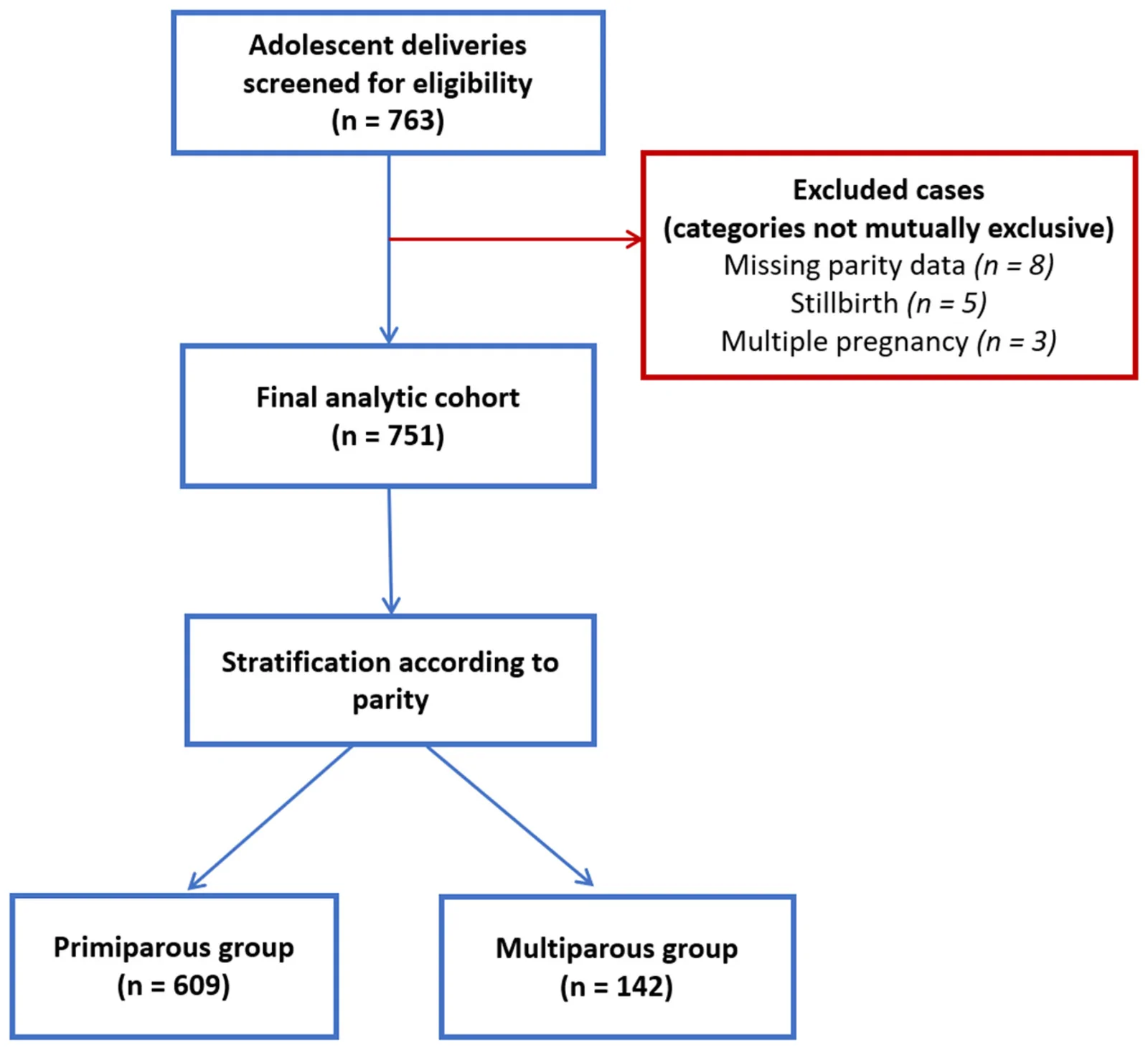
Flowchart of study population selection.

After applying the exclusion criteria, the final analytic cohort comprised 751 singleton live-birth deliveries to adolescent mothers eligible for analysis. Each included case represented one delivery episode recorded in the institutional birth registry. Parity was defined according to the maternal obstetric history at the time of the index delivery. Delivery episodes were classified according to maternal parity as primiparous (para 1) or multiparous (para ≥ 2). The study population selection process is illustrated in Figure 1.

Delivery episodes were classified according to maternal parity at the time of the index delivery as occurring in primiparous (para 1) or multiparous (para ≥ 2) adolescents.

### 2.2. Data Collection and Variables

A structured dataset was constructed from electronic medical records, including maternal, obstetric, and neonatal variables. Data extraction was performed retrospectively by three investigators using a standardized data collection form developed for the study. To minimize extraction errors and ensure data consistency, the resulting dataset was independently reviewed and verified by two clinicians experienced in managing underage deliveries, including one clinician practicing at the study hospital. When necessary, data were checked against the original electronic medical records. Records with incomplete or inconsistent information were reviewed before inclusion in the analytical dataset.

Maternal age was recorded in completed years and categorized as <15 versus ≥15 years for multivariable regression analyses, based on clinical relevance and the distribution observed in stratified analyses. Parity was defined as a binary variable (primiparous vs. multiparous). Mode of delivery was classified as vaginal or cesarean section. Gestational age was determined using first-trimester ultrasound when available or, alternatively, the date of the last menstrual period, and was analyzed as a continuous variable in models where it was not part of the outcome definition.

Neonatal outcomes included low birth weight (birth weight < 2500 g), preterm birth (delivery before 37 completed weeks of gestation), and Apgar score at 5 min, dichotomized as <7 versus ≥7.

In addition, a composite neonatal outcome was defined as the occurrence of at least one of the following: preterm birth, low birth weight, or Apgar score < 7 at 5 min.

### 2.3. Statistical Analysis

Statistical analyses were performed using JASP (version 0.19.3). Continuous variables were assessed for normality using the Shapiro–Wilk test and are presented as mean ± standard deviation or median (interquartile range), as appropriate. Categorical variables are reported as frequencies and percentages.

Comparisons between primiparous and multiparous adolescents were performed using the Mann–Whitney U test for continuous variables and the χ^2^ test for categorical variables. Effect sizes were reported using rank-biserial correlation for continuous variables and Cramer’s V for categorical variables.

To evaluate the independent association between parity and neonatal outcomes, multivariable logistic regression models were constructed for each outcome of interest. Parity was included in all models as the primary exposure variable. Covariates were selected a priori based on clinical relevance and evidence from previous literature regarding determinants of neonatal outcomes in adolescent pregnancies. Maternal age was included in all models as a clinically relevant covariate and was modeled categorically as <15 versus ≥15 years. Mode of delivery was included where clinically appropriate, whereas gestational age was included when appropriate for the outcome under investigation. Gestational age was not included in models where it was directly incorporated into the outcome definition (e.g., preterm birth and the composite outcome). All selected variables were entered simultaneously into the regression models rather than via automated variable selection procedures. An interaction term between maternal age and parity was initially tested and subsequently excluded from the final models due to lack of statistical significance, in accordance with the principle of model parsimony, which favors simpler models with comparable explanatory performance [17].

Predicted probabilities and corresponding 95% confidence intervals were generated from the final multivariable logistic regression models using model-based estimates. For graphical presentation, the predictor of interest was varied while the remaining covariates were held constant at their reference category or mean value, as appropriate.

Missing data were handled using complete-case analysis. For each outcome, only observations with available data for the outcome and corresponding covariates were included in the respective regression model. No imputation procedures were performed.

Multicollinearity among predictors was assessed using variance inflation factors (VIFs). Model performance was evaluated using likelihood-ratio statistics, pseudo-R^2^ measures (McFadden, Cox–Snell, and Nagelkerke), and the area under the receiver operating characteristic curve (AUC). Across all regression models, VIF values ranged from 1.00 to 1.04, indicating no evidence of problematic multicollinearity.

Adjusted odds ratios (aORs) with 95% confidence intervals (CIs) were reported. Statistical significance was set at *p* < 0.05.

## 3. Results

### 3.1. Cohort Characteristics by Parity

A total of 751 singleton live-birth deliveries to adolescent mothers met the inclusion criteria and were included in the final analysis. Most delivery episodes involved primiparous adolescents (n = 609, 81.1%), while 142 (18.9%) involved multiparous adolescents.

Maternal age differed significantly between groups, with multiparous adolescents being older than primiparous adolescents (*p* < 0.001). This difference was accompanied by a small effect size approaching the moderate threshold (rank-biserial correlation = 0.296), and age-group distribution also differed significantly between parity groups (Cramer’s V = 0.193). In contrast, rural residence, gestational age, birth weight, fetal presentation, and mode of delivery showed no significant between-group differences and were associated with negligible effect sizes (Table 1). Overall, maternal and obstetric characteristics were largely comparable between groups, with the exception of maternal age.

**Figure 2:**
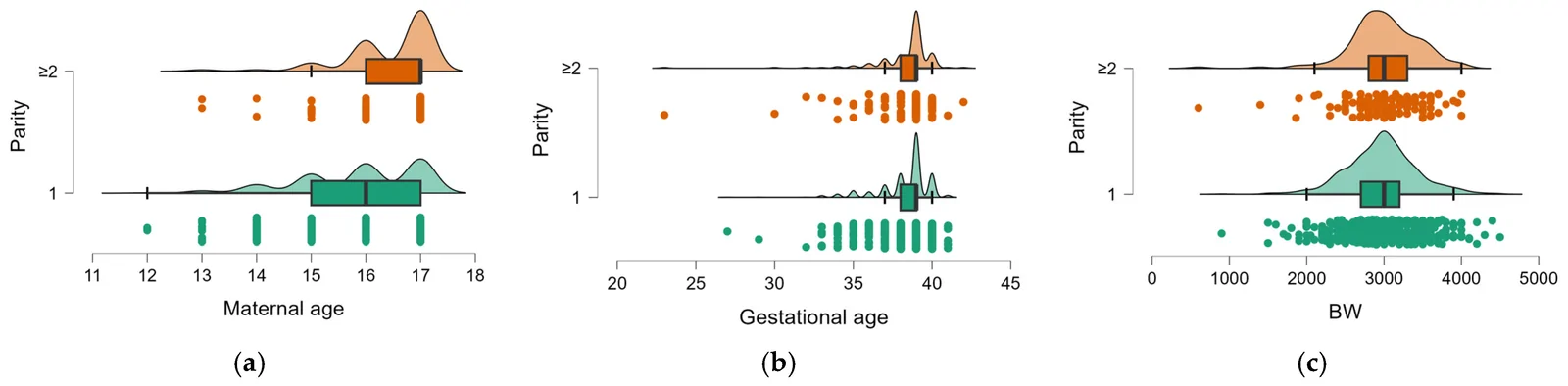
Distribution of maternal age (**a**), gestational age (**b**), and birth weight (**c**) according to parity. Boxplots represent the median and interquartile range, individual observations are shown as jittered points, and density curves illustrate the distribution of the data.

The distributions of maternal age, gestational age, and birth weight by parity are shown in Figure 2.

### 3.2. Neonatal Outcomes by Parity

The prevalence of low birth weight (<2500 g) was higher among primiparous adolescents than among multiparous adolescents (12.7% vs. 6.6%, *p* = 0.045). To further characterize the severity of prematurity, preterm births were stratified into gestational age categories. Most preterm births occurred in the late-preterm category, accounting for 74 of 88 preterm deliveries (84.1%), whereas more severe forms of prematurity were uncommon. No significant differences were observed between parity groups for preterm birth, prematurity categories, or Apgar score < 7 at 5 min (Table 2). Effect size estimates were small or negligible, indicating limited between-group differences in neonatal outcomes.

Stratified analyses were performed to further explore factors associated with Apgar score < 7 at 5 min (Table 3). The proportion of neonates with an Apgar score < 7 at 5 min was significantly higher among mothers younger than 15 years compared with those aged ≥15 years (10.1% vs. 4.1%, *p* = 0.025), whereas no significant differences were observed according to mode of delivery (*p* = 0.396). Similar findings were observed in parity-stratified analyses, with a higher frequency of Apgar score < 7 among primiparous adolescents younger than 15 years. Sensitivity analyses excluding mothers younger than 15 years confirmed the lack of association between parity and Apgar score < 7 at 5 min (*p* = 0.676; Cramer’s V = 0.017).

### 3.3. Multivariable Logistic Regression Analysis

To evaluate whether parity was independently associated with neonatal outcomes among adolescent mothers, separate multivariable logistic regression models were constructed for Apgar score < 7 at 5 min, preterm birth, low birth weight, and a composite adverse neonatal outcome. Model performance varied across outcomes. The low birth weight model demonstrated the best overall fit and discrimination (Nagelkerke R^2^ = 0.244; AUC = 0.763), whereas the models for Apgar score < 7 at 5 min, preterm birth, and the composite adverse neonatal outcome showed limited explanatory power and weak discrimination (Nagelkerke R^2^ range: 0.001–0.044; AUC range: 0.515–0.574). No evidence of problematic multicollinearity was identified in any model (VIF range: 1.00–1.04).

#### 3.3.1. Apgar Score < 7 at 5 min

To account for the potential non-linear effect suggested by the stratified analyses, maternal age was modeled as a categorical variable (<15 vs. ≥15 years). In the adjusted model, maternal age < 15 years was associated with higher odds of Apgar score < 7 at 5 min (aOR 2.76, 95% CI 1.12–6.76, *p* = 0.027). Gestational age was also associated with the outcome, with each additional week corresponding to lower odds of Apgar score < 7 (aOR 0.83, 95% CI 0.72–0.97, *p* = 0.019). Multiparity and mode of delivery were not significantly associated with Apgar score < 7 at 5 min (Table 4).

**Figure 3:**
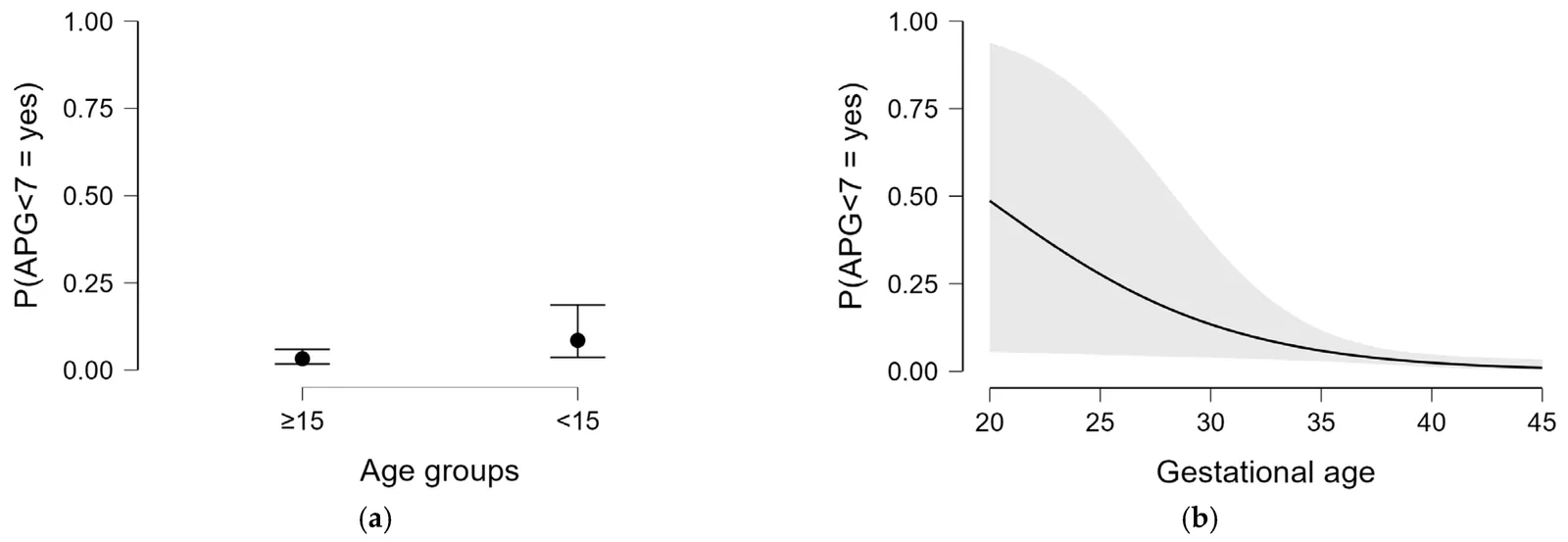
Model-based predicted probability of Apgar score < 7 at 5 min derived from the adjusted logistic regression model according to maternal age group (**a**) and gestational age (**b**). For each plot, the predictor of interest was varied while the remaining covariates were held constant at their reference categories: primiparity and cesarean delivery; gestational age was held at its mean value for panel (**a**), and maternal age group was held at ≥15 years for panel (**b**). Shaded areas represent 95% confidence intervals.

As illustrated in Figure 3, younger maternal age (<15 years) was associated with a higher predicted probability of Apgar score < 7, while increasing gestational age was associated with a progressive decrease in risk.

#### 3.3.2. Preterm Birth

In the adjusted model, multiparity was not significantly associated with preterm birth (aOR 0.86, 95% CI 0.48–1.56, *p* = 0.622). Maternal age < 15 years was also not associated with preterm birth (aOR 1.14, 95% CI 0.56–2.33, *p* = 0.713) (Table 5). The model showed very limited explanatory power and weak discrimination.

#### 3.3.3. Low Birth Weight

In the adjusted model (Table 6), multiparity was associated with lower odds of low birth weight (aOR 0.40, 95% CI 0.17–0.92, *p* = 0.031). Gestational age was strongly associated with the outcome, with each additional week corresponding to lower odds of low birth weight (aOR 0.56, 95% CI 0.50–0.65, *p* < 0.001). Maternal age < 15 years and mode of delivery were not significantly associated with low birth weight. 

**Figure 4:**
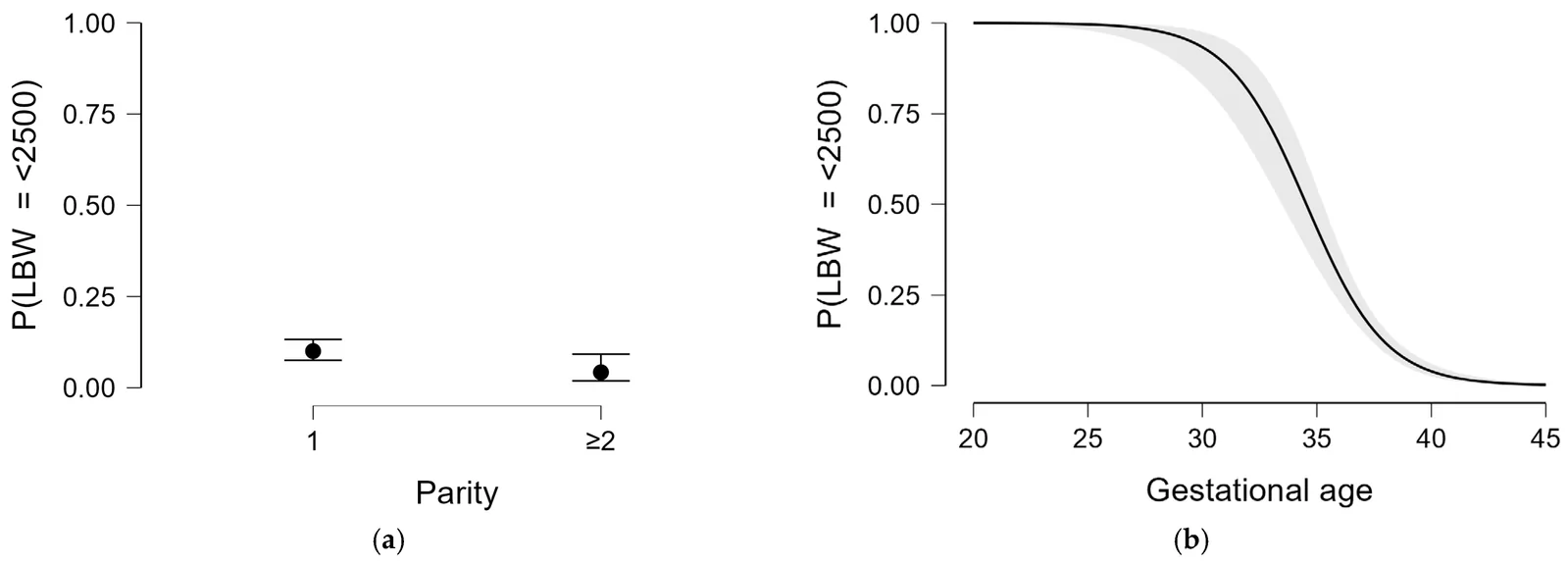
Model-based predicted probability of low birth weight derived from the adjusted logistic regression model according to parity (**a**) and gestational age (**b**). For each plot, the predictor of interest was varied while the remaining covariates were held constant at their reference categories or mean values, as appropriate. Shaded areas represent 95% confidence intervals.

The predicted probability of low birth weight by parity and gestational age is shown in Figure 4.

#### 3.3.4. Composite Adverse Neonatal Outcome

A composite adverse neonatal outcome was defined as the occurrence of at least one of the following: preterm birth, low birth weight, or Apgar score < 7 at 5 min.

Multiparity was not significantly associated with the composite adverse neonatal outcome (aOR 0.64, 95% CI 0.39–1.07, *p* = 0.088). Maternal age < 15 years showed a non-significant trend toward higher odds of the composite outcome (aOR 1.64, 95% CI 0.96–2.81, *p* = 0.069), while vaginal delivery was not significantly associated with the outcome (aOR 1.24, 95% CI 0.87–1.79, *p* = 0.236) (Table 7).

These results indicate that parity was not consistently associated with adverse neonatal outcomes across endpoints. Multiparity was associated only with lower odds of low birth weight after adjustment for gestational age, whereas maternal age < 15 years and gestational age were more relevant for Apgar score < 7 at 5 min.

## 4. Discussion

Adolescent pregnancy is associated with an increased risk of adverse neonatal outcomes, including low birth weight and preterm birth, as consistently demonstrated in large population-based and multicountry studies [3,4]. These risks reflect a complex interplay between biological immaturity and social vulnerability, the relative contribution of which varies across healthcare settings [7,9]. In this study, we evaluated the independent association between parity and neonatal outcomes within this high-risk population. Our findings suggest that parity was not associated with preterm birth or an Apgar score < 7 at 5 min, whereas multiparity was associated with lower odds of low birth weight after adjustment for gestational age. However, given the observational design and the possibility of residual confounding, this finding should be considered hypothesis-generating and interpreted cautiously. Overall, the associations observed suggest that the relationship between parity and neonatal outcomes in adolescent pregnancies may be heterogeneous and outcome-specific.

The absence of an association between parity and preterm birth in our cohort is consistent with evidence indicating that prematurity in adolescent populations is primarily driven by biological and contextual determinants rather than obstetric history [4,10,18]. Several large-scale analyses have shown that the increased risk of preterm birth among adolescents is substantially attenuated after adjustment for socioeconomic status, access to prenatal care, and maternal education [18]. Furthermore, younger maternal age, particularly below 15–16 years, has been identified as a stronger determinant of prematurity and neonatal mortality than parity itself [5,9]. Our findings are consistent with the interpretation that parity alone may not be a major determinant of preterm birth among adolescent mothers, although unmeasured biological and contextual factors may still contribute to gestational duration in this population.

Maternal age < 15 years was associated with higher odds of an Apgar score < 7 at 5 min in the adjusted model, whereas gestational age was associated with lower odds of this outcome, underscoring the role of both biological immaturity and gestational maturity in early neonatal adaptation. Previous studies have shown that Apgar score is strongly influenced by gestational age and perinatal conditions [19,20], while younger maternal age has been linked to increased vulnerability during labor and delivery, potentially contributing to compromised neonatal condition at birth. The absence of a significant association with parity further supports the notion that early neonatal adaptation is not substantially influenced by prior reproductive experience in adolescent populations.

In contrast, the observed association between multiparity and lower odds of low birth weight represents a notable finding of this study. This association contrasts with unadjusted analyses in our cohort, highlighting the role of confounding, particularly gestational age, in shaping the relationship between parity and birth weight. Previous studies have reported heterogeneous findings, with some indicating increased risks in repeat adolescent pregnancies, often attributed to socioeconomic disadvantage and short interpregnancy intervals [13,21,22], while others have found no independent effect after adjustment [23]. Our results support the possibility that the association between parity and fetal growth is highly context-dependent.

From a clinical perspective, the observed associations appear modest despite their statistical significance. While multiparity was associated with lower odds of low birth weight, effect size estimates indicated only limited between-group differences, and parity was not consistently associated with the other neonatal outcomes evaluated, including the composite adverse neonatal outcome. Taken together, these findings suggest that parity alone may have limited clinical utility for risk stratification in adolescent pregnancies compared with factors such as gestational age and maternal age.

In addition, differential patterns of fetal growth versus prematurity in adolescent populations suggest that these outcomes may arise from partially distinct biological and environmental mechanisms, supporting their evaluation as separate dimensions of neonatal risk rather than interchangeable markers [7,24].

The mechanisms underlying the observed association between multiparity and lower odds of low birth weight cannot be determined from the present study. Therefore, any discussion of potential biological pathways should be considered exploratory and based on evidence from previous literature rather than on direct observations from our data.

The analysis of the composite adverse neonatal outcome did not show a significant association with parity. Although the point estimate for multiparity was below unity, the confidence interval crossed the null value, indicating that this finding should not be interpreted as evidence of a protective association. Maternal age < 15 years showed only a non-significant trend toward higher odds of the composite outcome, suggesting that its association may be more apparent for early neonatal adaptation, as reflected by Apgar score < 7, than for the broader composite endpoint.

Previous studies have suggested that differences in uteroplacental vascular adaptation across successive pregnancies may influence fetal growth, including potential changes in uteroplacental blood flow resistance and maternal physiological adaptation [8,13]. However, the relevance of these mechanisms in adolescent populations remains uncertain. Adolescents are still undergoing somatic growth, and competition for nutritional resources between maternal and fetal compartments may modify these adaptive processes [11,12]. Furthermore, important contextual factors such as socioeconomic conditions, nutritional status, interpregnancy interval, and adequacy of prenatal care were not available in the present study and may have contributed to the observed association. Therefore, the association between multiparity and lower odds of low birth weight should be interpreted cautiously and considered hypothesis-generating rather than evidence of a protective effect or biological advantage of multiparity.

At the same time, the apparent association observed in our cohort may reflect a selection effect rather than a direct causal relationship. Adolescents who experience repeat pregnancies may represent a subgroup with distinct biological or social characteristics, potentially including differences in physiological adaptation or access to care. This selection process may operate in both directions, as repeat adolescent pregnancies may occur either in highly vulnerable populations or, conversely, among individuals with greater biological or contextual adaptation to pregnancy [13,23].

The divergence between neonatal outcomes observed in this study highlights the complexity of risk pathways in adolescent pregnancies. While preterm birth and early neonatal adaptation may reflect different dimensions of gestational maturity and perinatal adaptation, fetal growth may be more sensitive to cumulative maternal, environmental, and socioeconomic influences. This distinction is consistent with established epidemiological frameworks differentiating the etiological pathways of prematurity and fetal growth restriction, which emphasize the role of placental perfusion and vascular adaptation in determining fetal growth trajectories [7,8].

Previous Romanian studies have similarly emphasized the multifactorial nature of pregnancy-related risks, highlighting the contribution of biological, obstetric, and contextual factors to maternal and neonatal outcomes [25,26]. In addition, our previous work examining obstetric outcomes among underage mothers in Romania demonstrated significant variability in maternal and neonatal outcomes according to age subgroup, further supporting the view that adolescent pregnancy represents a heterogeneous clinical entity rather than a single risk category [27].

The clinical context of this study is also relevant for interpretation. Conducted in a secondary-care hospital with standardized obstetric management and routine social and psychological counseling for underage mothers, the study environment likely reduced variability in care and provided a structured support framework for adolescent pregnancies. Previous research has shown that access to comprehensive antenatal care can attenuate disparities in neonatal outcomes among young mothers [3,4,28]. These results also have implications at the health system level, as they suggest that disparities in neonatal outcomes among adolescent mothers may be partly modifiable through structured prenatal care, standardized clinical management, and psychosocial support, rather than being determined solely by biological risk factors.

In the context of a secondary-care hospital providing standardized obstetric care and routine psychosocial counseling for minors, parity alone appears to have limited utility for neonatal risk stratification. However, its clinical relevance may differ across healthcare settings with varying patient populations, healthcare resources, psychosocial support services, and access to prenatal care. Therefore, caution is warranted when extrapolating these findings to other healthcare systems.

Several limitations should be acknowledged. The retrospective observational design precludes causal inference, and all reported associations should therefore be interpreted as observational rather than causal relationships. In addition, the relatively small number of adverse events, particularly low Apgar scores and very preterm births, may have reduced statistical power and limited the precision of some regression estimates. Five stillbirths were identified among the 763 adolescent deliveries initially screened, corresponding to a stillbirth proportion of 0.66% (6.6 per 1000 deliveries). These cases were excluded because the present study focused on neonatal outcomes among live-born infants. Although the small number of events precluded further analysis, the occurrence of stillbirths in this adolescent cohort underscores the importance of continued monitoring of severe perinatal outcomes in this vulnerable population.

Several potentially important confounding variables, including socioeconomic status, adequacy of prenatal care, nutritional status, smoking, substance use, interpregnancy interval, and individual-level psychosocial support, were not available in the dataset. These factors may influence both parity and neonatal outcomes, and residual confounding therefore cannot be excluded. Among these variables, the absence of information on interpregnancy interval is particularly relevant, as its association with adverse neonatal outcomes is complex and may be partly explained by unmeasured maternal characteristics rather than a direct causal effect [22,29].

Furthermore, parity was analyzed as a binary variable (primiparous versus multiparous), which may oversimplify reproductive history and does not account for gravidity, previous pregnancy losses, or the interval between pregnancies. Missing data were present for several variables and outcomes, resulting in varying denominators across analyses. Although complete-case analysis was used, the potential influence of missing data on the observed associations cannot be excluded.

As a single-center study, the findings may not be generalizable to other populations with different socioeconomic or healthcare contexts. The observed associations may differ in settings with limited access to prenatal care or greater socioeconomic disparities, where contextual factors may exert a stronger influence on neonatal outcomes. The possibility of selection bias should also be considered. Adolescents delivering in a secondary-care hospital with structured obstetric and psychosocial support services may differ systematically from those delivering in smaller hospitals or those with limited access to healthcare services, potentially influencing the observed outcome patterns. In addition, the small number of multiparous adolescents younger than 15 years limited the ability to perform reliable subgroup analyses and precluded robust assessment of potential interactions between maternal age and parity. These limitations should be considered when interpreting the observed associations, particularly those involving low-frequency outcomes and parity-related differences.

Future prospective multicenter studies incorporating detailed measures of socioeconomic status, interpregnancy interval, nutritional status, smoking, substance use, psychosocial support, and access to antenatal care are needed to further clarify the relative contributions of biological, reproductive, and contextual factors to neonatal outcomes in adolescent pregnancies.

Despite these limitations, the study has several strengths. It included a relatively large cohort of 751 adolescent singleton live-birth deliveries, representing one of the larger single-center studies specifically designed to evaluate the independent association between parity and neonatal outcomes in adolescent pregnancies. Multiple clinically relevant neonatal outcomes, including low birth weight, preterm birth, Apgar score < 7 at 5 min, and a composite adverse neonatal outcome, were evaluated using adjusted multivariable regression models, allowing assessment beyond simple univariate comparisons.

The study also provides evidence from an Eastern European setting, a region that remains comparatively underrepresented in the literature on adolescent pregnancy despite persistently high adolescent birth rates. In addition, sensitivity analyses performed for the Apgar outcome yielded findings consistent with the primary analyses, supporting the robustness of the observed associations. Finally, the finding that the association with parity was observed for low birth weight but not for preterm birth, Apgar score < 7, or the composite outcome contributes to a more nuanced understanding of adolescent pregnancy and highlights the need for refined, context-specific risk assessment approaches.

## 5. Conclusions

The findings of this study suggest that, within this cohort of adolescent pregnancies, parity alone appeared to have limited clinical utility for neonatal risk stratification. Parity was not significantly associated with preterm birth, Apgar score < 7 at 5 min, or the composite adverse neonatal outcome, while multiparity was associated with lower odds of low birth weight. This finding should be interpreted cautiously because residual confounding cannot be excluded. Future prospective multicenter studies incorporating socioeconomic, reproductive, and healthcare-related determinants are needed to further clarify the relationship between parity and neonatal outcomes in adolescent populations.

## Figures and Tables

**Table 1:** Maternal and obstetric characteristics according to parity.

Variable	Primiparous (n = 609)	Multiparous (n = 142)	*p*	Effect Size
Maternal age (years), median (IQR)	16 (15–17)	17 (16–17)	<0.001	rrb = 0.296
Age group, n (%)			<0.001	V = 0.193
<15 years	74 (12.2%)	4 (2.8%)		
15–16 years	315 (51.7%)	55 (38.7%)		
17 years	220 (36.1%)	83 (58.5%)		
Rural residence, n (%)	376 (61.7%)	80 (56.3%)	0.235	V = 0.043
Gestational age (weeks), median (IQR) *	39 (38–39)	39 (38–39)	0.632	rrb = 0.025
Birth weight (g), median (IQR) *	3000 (2700–3200)	3000 (2800–3300)	0.289	rrb = 0.058
Non-cephalic presentation, n/N (%) **	21/598 (3.5%)	3/137 (2.2%)	0.328	V = 0.055
Cesarean delivery, n (%)	294 (48.3%)	68 (47.9%)	0.934	V = 0.003

* Based on available data; ** Excluding missing/unspecified presentation. IQR, interquartile range; rrb, rank-biserial correlation; V, Cramer’s V. Effect sizes were interpreted as negligible (<0.10), small (0.10–0.29), moderate (0.30–0.49), and large (≥0.50).

**Table 2:** Neonatal outcomes according to parity.

Outcome	Primiparous n/N (%)	Multiparous n/N (%)	*p*	Effect Size
Low birth weight (<2500 g)	73/577 (12.7%)	9/137 (6.6%)	0.045	V = 0.075
Preterm birth (<37 weeks)	73/578 (12.6%)	15/137 (10.9%)	0.590	V = 0.020
Prematurity categories			0.497 *	V = 0.069
Extremely preterm (<28 weeks)	1/578 (0.2%)	1/137 (0.7%)		
Very preterm (28–31 weeks)	1/578 (0.2%)	1/137 (0.7%)		
Moderate preterm (32–33 weeks)	8/578 (1.4%)	2/137 (1.5%)		
Late preterm (34–36 weeks)	63/578 (10.9%)	11/137 (8.0%)		
Apgar score < 7	26/548 (4.7%)	6/130 (4.6%)	0.95	V = 0.002

* *p*-value derived from the overall comparison of gestational-age category distribution between parity groups (χ^2^ test).

**Table 3:** Apgar score < 7 at 5 min according to maternal age group and delivery.

Variable	Apgar < 7 n/N (%)	*p*-Value
Maternal age group		0.025
<15 years	7/69 (10.1%)	
≥15 years	25/609 (4.1%)	
Mode of delivery		0.396
Cesarean delivery	13/325 (4.0%)	
Vaginal delivery	19/353 (5.4%)	
Parity-stratified analysis (primiparous)		0.015
<15 years	7/58 (10.8%)	
≥15 years	19/463 (3.9%)	
Parity-stratified analysis (multiparous)		0.655
<15 years	0/4 (0.00%)	
≥15 years	6/120 (4.8%)	

**Table 4:** Multivariable logistic regression model for Apgar score < 7 at 5 min.

Variable	aOR	95% CI	*p*
Multiparity (≥2 vs. primiparity)	1.03	0.40–2.65	0.945
Maternal age (<15 vs. ≥15 years)	2.76	1.12–6.76	0.027
Gestational age (per week increase)	0.83	0.72–0.97	0.019
Vaginal delivery (vs. cesarean)	1.38	0.66–2.85	0.391

Model performance: LR χ^2^ = 9.49 (*p* = 0.050); McFadden R^2^ = 0.037; Nagelkerke R^2^ = 0.044; Cox–Snell R^2^ = 0.014; AUC = 0.574, indicating weak discrimination. No evidence of problematic multicollinearity was identified (VIF range: 1.00–1.04). Model based on 677 observations. aOR, adjusted odds ratio; CI, confidence interval.

**Table 5:** Multivariable logistic regression model for preterm birth.

Variable	aOR	95% CI	*p*
Multiparity (≥2 vs. primiparity)	0.86	0.48–1.56	0.622
Maternal age (<15 vs. ≥15 years)	1.14	0.56–2.33	0.713

Model performance: LR χ^2^ = 0.43 (*p* = 0.807); McFadden R^2^ = 0.001; Nagelkerke R^2^ = 0.001; Cox–Snell R^2^ = 0.001; AUC = 0.515, indicating weak discrimination. No evidence of problematic multicollinearity was identified (VIF = 1.013). Model based on 715 observations. aOR, adjusted odds ratio; CI, confidence interval.

**Table 6:** Multivariable logistic regression model for low birth weight (<2500 g).

Variable	aOR	95% CI	*p*-Value
Multiparity (≥2 vs. primiparity)	0.40	0.17–0.92	0.031
Gestational age (per week increase)	0.56	0.50–0.65	<0.001
Maternal age (<15 vs. ≥15 years)	0.99	0.44–2.24	0.979
Vaginal delivery (vs. cesarean)	1.08	0.65–1.81	0.765

Model performance: LR χ^2^ = 94.20 (*p* < 0.001); McFadden R^2^ = 0.187; Nagelkerke R^2^ = 0.244; Cox–Snell R^2^ = 0.124; AUC = 0.763, indicating acceptable discrimination. No evidence of problematic multicollinearity was identified (VIF range: 1.00–1.01). Model based on 713 observations. aOR, adjusted odds ratio; CI, confidence interval.

**Table 7:** Multivariable logistic regression model for composite adverse neonatal outcome.

Variable	aOR	95% CI	*p*-Value
Multiparity (≥2 vs. primiparity)	0.64	0.39–1.07	0.088
Maternal age (<15 vs. ≥15 years)	1.64	0.96–2.81	0.069
Vaginal delivery (vs. cesarean)	1.24	0.87–1.79	0.236

Model performance: LR χ^2^ = 8.48 (*p* = 0.037); McFadden R^2^ = 0.011; Nagelkerke R^2^ = 0.019; Cox–Snell R^2^ = 0.012; AUC = 0.569, indicating weak discrimination. No evidence of problematic multicollinearity was identified (VIF range: 1.00–1.01). Model based on 695 observations. aOR, adjusted odds ratio; CI, confidence interval.

## Data Availability

The original contributions presented in this study are included in the article. Further inquiries can be directed to the corresponding author.

## Abbreviations

The following abbreviations are used in this manuscript:

aOR	Adjusted odds ratio
CI	Confidence interval
LBW	Low birth weight
